# Evaluating the association of adverse childhood experiences, mood and anxiety disorders, and suicidal ideation among behavioral health patients at a large federally qualified health center

**DOI:** 10.1371/journal.pone.0254385

**Published:** 2021-07-12

**Authors:** April Joy Damian, May Oo, Daniel Bryant, Joseph J. Gallo

**Affiliations:** 1 Weitzman Institute, Community Health Center, Inc., Middletown, Connecticut, United States of America; 2 Department of Mental Health, Johns Hopkins University Bloomberg School of Public Health, Baltimore, Maryland, United States of America; Michigan State University College of Human Medicine, UNITED STATES

## Abstract

Although numerous studies have examined the relationship between adverse childhood experiences (ACEs) and suicide, research is needed that studies the effects of specific ACEs, including subclasses of ACEs, independent of the effect of other ACEs. We explored the relationship between ACEs and suicidal ideation (SI) with special attention to patterns according to generation cohort (millennials: 22–37 years old, generation X: 38–53 years old, and baby boomers: 54–72 years old), and assessed the extent to which mood and anxiety disorders account for the relationship between ACEs and suicidal ideation. Patients in behavioral healthcare services of a large federally qualified health center (n = 4,392) were assessed at baseline on ACEs, SI, and mood and anxiety disorders. Logistic regression modeling was used to examine the data. Emotional neglect was the strongest predictor of SI among Millennials (OR = 1.59), Generation X (OR = 1.81), and Baby Boomers (OR = 1.88) after controlling for mood and anxiety disorders, race/ethnicity, and gender. Findings suggest ACEs predict an increased likelihood of having SI over and above the influence of mood and anxiety disorders, in models adjusted for gender and race/ethnicity. Across generations, the association with suicidal ideation was strongest for any child abuse and neglect, but not for household dysfunction. The observed association of ACEs with suicidal ideation suggests that ACEs should be considered as a risk factor and incorporated into screening assessments for suicidal ideation. Lastly, additional research on the association of ACEs and suicidality in individuals not actively being managed in behavioral healthcare settings is also warranted.

## 1. Introduction

Suicide remains a significant public health challenge that continues to rise at record rates in the United States and is at the highest level since World War II [1]. While the causes of suicide are complex and multifactorial, a large body of literature examines potential antecedents and risk factors of this epidemic, including mood and anxiety disorders and adverse childhood experiences. Depression has been identified as the leading risk factor for suicide [2–4]. Adverse childhood experiences (ACEs) generally refer to potentially traumatic events occurring before the age of 18 years [5]. Although various approaches have been used to define and assess ACEs, studies have consistently supported a strong graded relationship between ACEs and suicide-related behaviors, including suicidal ideation and attempts [6–8]. Research to date has also pointed out the association of suicide with specific ACEs; sexual and emotional abuse, neglect, and exposure to domestic violence in particular, have been found to increase the likelihood of suicidal behavior (i.e., attempts, ideation) compared to other ACEs [2, 6, 8–10].

Additionally, it is important to highlight disparities in suicide and how certain populations are disproportionately affected by this public health problem. According to the Centers for Disease Control and Prevention (CDC) National Center for Health Statistics, while suicide ranks as the 10^th^ leading cause of death nationwide, the phenomenon has become more prominent in certain age groups in recent years: suicide is now the second leading cause of death for ages 10–34 and the fourth leading cause for ages 35–54 [11]. For men, the rate increased 26% from 17.8 per 100,000 in 1999 to 22.4 per 100,000 in 2017, while the rate for women increased 43% from 4.0 to 6.1 over the same period [1]. In addition, from 2015 to 2016, the age-adjusted suicide rate for the total U.S. population increased from 13.3 per 100,000 standard population to 13.5 (an increase of 1.5%). The rate increased from 5.8 to 6.3 (8.6%) for non-Hispanic blacks and from 6.2 to 6.7 (8.1%) for Hispanics; it remained unchanged for non-Hispanic whites [12].

Consequently, the purpose of the current study was to explore three questions. **The first question examines whether there is a relationship between specific ACEs and suicidal ideation among adults**. Although there have been several studies on the relationship between suicide and mood and anxiety disorders, suicide and ACEs, as well as mood and anxiety disorders and their association with ACEs [6, 8, 13–15], there is a paucity of literature on the association of suicide, mood and anxiety disorders, and ACEs. In addition, while several studies have examined the role of specific ACEs and their association with negative health-related sequelae, including suicide and mood and anxiety disorders, such studies have either not considered the role of multiple ACEs or not considered associations independent of other ACEs. Similarly, less is known about the association of *subclasses* of ACEs and the degree to which the likelihood of suicide differs among those who have experienced child abuse and neglect only (e.g., emotional, verbal, physical abuse and neglect) compared to those who have solely experienced household dysfunction (e.g., divorce, witnessing violence in the household) in childhood. Thus, there is a need for research that examines the effects of specific ACEs, including subclasses of ACEs, independent of the effect of other ACEs.

**The second question examines the extent to which the relationship between ACEs and suicidal ideation varies by generational cohort affiliation**. Although there is a growing body of literature examining this relationship based on gender and sexual orientation as well as race and ethnicity, there are major gaps in the literature on suicide and risk factors based on age/generation cohort, which is of particular importance given the unprecedented disparities in suicide observed based on these demographic factors in recent years [7, 8, 13]. A recent report highlighting alcohol and drug misuse, suicide, collectively referred to as deaths of despair, noted that there were substantial differences in prevalence based on generation cohort. For example, for millennials, there was a 35% increase in suicide deaths over the past decade; in contrast, for Generation X and baby boomers, suicide rates increased by 14% and 24%, respectively, during the same timeframe [16].

**The third question explores the extent to which ACEs are associated with suicidal ideation independent of mood and anxiety disorders**. Prior research has suggested that there is a significant relationship between ACEs and suicidal ideation, over and above that of the association of mood and anxiety disorders with suicidal ideation [8, 13, 17, 18]. More than 30% of adult mental health challenges, including mood and anxiety disorders and suicidal ideation, are attributable to childhood adversity [13, 14]. Biological, psychological, and social pathways may underlie the connections between ACEs, mood and anxiety disorders, and suicidal ideation. ACEs may set the stage for biological changes in the brain that increase vulnerability to mental disorders and self-destructive behavior in adulthood [18, 19]. Emotional traumatic experiences in childhood can have a negative influence on self perceptions and self-esteem, increasing the potential for depression and anxiety [20, 21]. Lack of social support and diminished social connectedness may play a role in the development of mood and anxiety disorders [20–22]. Since ACEs often are not acknowledged or addressed in health care, the adverse influence of ACEs on mental health and suicidality may be perpetuated across the life course [23].

**Fig 1:**
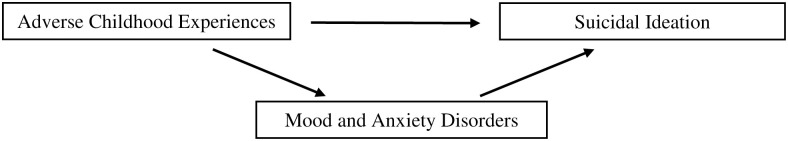
Association between suicidal ideation, mood and anxiety disorders, and adverse childhood experiences.

Our study explores the relationship between ACEs and suicidal ideation, including subclasses and individual types of ACEs, with special attention to patterns according to generation cohort, and assessing the extent to which mood and anxiety disorders account for the relationship between ACEs and suicidal ideation (Fig 1).

## 2. Methods

### 2.1. Sample

The sample consisted of patients in behavioral healthcare services between March 2017 and January 2019 at Community Health Center Inc. (CHC), a large federally qualified health center (FQHC) in Connecticut. The behavioral healthcare programs serve individuals with social and medical complexity. All of the patients with behavioral health needs were referred to CHC for comprehensive behavioral health services by their primary care provider. In all, 4,748 individuals completed a semi-structured intake developed by the FQHC. The semi-structured intake contains questions on presenting concern, symptoms clusters. The purpose of a semi-structured intake assessment was to identify diagnoses, maintain compliance with regulations related to the health and safety of patients, and improve care for patients enrolled in behavioral healthcare services at CHC. The ACEs data were collected from the intake assessment. Patients who did not have a complete set of ACE data were excluded, resulting in a total of 4,392 unique individuals who participated in the study (92.5% of the eligible population). All the data in this retrospective study was fully anonymized before any members of the research team were able to access the data. CHC’s institutional review board approved this study under exemption categories and waived the requirement for informed consent (45 CFR 46.101(b)(4)).

### 2.2. Measures

The 10-item Adverse Childhood Experiences survey [24] was used. The survey assesses childhood abuse/neglect and household dysfunction. Verbal abuse, physical abuse, sexual abuse, emotional neglect, physical neglect are defined as childhood abuse and neglect. Household dysfunction includes parental separation, witnessing domestic violence, household substance abuse, household mental illness or suicidal ideation, and household incarceration. Events were scored as ‘0’ if the individual did not experience them and ‘1’ if they did. Later, scores were summed to reflect the cumulative exposure to ACEs [25–27]. Age, race/ethnicity, and gender collected as a part of routine care were used in statistical analyses.

Suicidality was assessed using the 9^th^ question of the Patient Health Questionnaire 9 (PHQ-9) [28, 29]. Recent studies conducted in outpatient settings [30] have found suicidal ideation, as assessed by item 9 of the PHQ9, is a robust predictor of suicide attempts and deaths regardless of age and inclusive of racially/ethnically diverse populations. The PHQ-9 is administered in compliance with the Uniform Data System (UDS) measures on an annual basis to all patients at CHC who screen positive with the Patient Health Questionnaire 2 (PHQ-2). The 9^th^ question asks whether, in the past two weeks, the patient has had “Thoughts that [they] would be better off dead, or thoughts of hurting [themselves] in some way?” The potential answers are “Not at all,” “Several days,” “More than half the days,” or “Nearly every day.” Any response greater than “Not at all” across all historical screenings was treated as a being positive for suicidality.

Mood and anxiety disorders were assessed as a part of the semi-structured intake interview when patients enter treatment. All diagnoses were arrived at by licensed independent providers using the DSM 5. For the purposes of this study we included the most prevalent mood and anxiety disorders among CHC adult behavioral health patients, specifically, Major Depressive Disorder, Bipolar I and Bipolar II disorder, and Generalized Anxiety Disorder.

### 2.3. Analysis

Descriptive statistics were summarized for the variables of interest. Comparisons between groups were performed using chi-squared test. The probability of suicidal ideation in subjects who reported exposure to at least one ACE was compared with that of those who had never exposed to ACEs (0 number of ACEs as reference), by calculating odds ratios (*OR*s) with 95% confidence intervals (*CI*s) using logistic regression models. Additionally, two composite measures made up of the individual child abuse and neglect (CAN) and household dysfunction (HD) type ACEs, respectively, were created to allow for analysis of independent effects of subclasses of ACEs. The analysis was stratified by generation (millennials: 22–37 years old, generation X: 38–53 years old, and baby boomers: 54–72 years old). Adjustments were made for mood and anxiety disorders, race/ethnicity, and gender in the analyses. An alpha level of *p* ≤0.05 was used to determine statistical significance for all analyses. All statistical procedures were conducted using SPSS (version 22).

## 3. Results

### 3.1. Demographic characteristics

Table 1 shows the prevalence of race/ethnicity, gender, the total number of Adverse Childhood Experiences (ACEs), type of ACE, lifetime mood and anxiety disorder, and history of suicidal ideation by age/generation cohort for the sample (*n* = 4,392). The sample consisted of 37.2% millennials (ages 22–37), 30.7% Generation X (ages 38–53), and 21.1% baby boomers (ages 54–72). The majority of the participants in all three different age groups identified themselves as Non-Hispanic White ranging from 45.3% to 55.5%. The study sample was predominantly female ranging from 55.1% to 60.6%. Each age/generation cohort experiences ACEs almost equally. An overwhelming majority (80.3% to 87.6%) of the total sample population reported at least one ACE and half of the sample total (42.1% to 52.8%) had 4 or more ACEs. Millennials had the highest prevalence of parental divorce (65.8%, *p* < .001), verbal abuse (49.9%, *p* = .002), household drug use (46.8%), household mental illness (46.3%, *p* < .001), emotional neglect (44.5%, *p* < .001), and household incarceration (25.6%, *p* < .001) compared to Generation X and baby boomers. However, the prevalence of physical abuse (40.9%, *p* = .030), sexual abuse (34.4%, *p* = .012), and witnessed intimate partner violence (30.4%, *p* = .005) were more common in Generation X than others. More than half of the sample (51.4%-63.9%) had exposure to both childhood abuse/neglect and household dysfunction. Of the 4,392 participants, 38.2%-47.7% had been diagnosed with the major depressive disorder, and 1 out of 5 individuals in the sample responded in the affirmative to PHQ9 item 9 about thoughts of being better off dead or hurting oneself in some way over the last two weeks.

**Table 1:** Sample characteristics (*n* = 4,392).

	Millennials (22–37 years) *n* (%)	Generation X (38–53 years) *n* (%)	Baby Boomers (54–72 years) *n* (%)	χ^2^ ^p^
**Race/Ethnicity**				
Non-Hispanic White	741 (45.3%)	626 (46.5%)	513 (55.5%)	19.32^.036^
Non-Hispanic Black	194 (11.9%)	173 (12.8%)	115 (12.4%)	
Hispanic/Latinx	370 (22.6%)	326 (24.2%)	183 (19.8%)	
Asian	15 (0.9%)	12 (0.9%)	6 (0.6%)	
American Indian/Pacific Islander	7 (0.4%)	4 (0.3%)	2 (0.2%)	
Other	41 (2.5%)	28 (2.1%)	15 (1.6%)	
**Gender**				
Male	643 (39.4%)	508 (37.7%)	415 (44.9%)	12.32^.002^
Female	991 (60.6%)	839 (62.3%)	510 (55.1%)	
**Total Number of Adverse Childhood Experiences (ACEs)**				
0	201 (12.3%)	210 (15.6%)	183 (19.8%)	50.37***
1	182 (11.1%)	164 (12.2%)	132 (14.3%)	
2	223 (13.6%)	139 (10.3%)	116 (12.5%)	
3	166 (10.1%)	164 (12.2%)	105 (11.4%)	
4+	864 (52.8%)	670 (49.7%)	389 (42.1%)	
**ACE Type**				
***Child Abuse and Neglect (CAN)***				
Verbal Abuse	816 (49.9%)	642 (47.7%)	394 (42.6%)	12.63^.002^
Physical Abuse	606 (37%)	551 (40.9%)	333 (36.0%)	7^.030^
Sexual Abuse	495 (30.3%)	463 (34.4%)	269 (29.1%)	8.83^.012^
Emotional Neglect	728 (44.5%)	559 (41.5%)	333 (36.0%)	17.49***
Physical Neglect	327 (20.0%)	270 (20.0%)	169 (18.3%)	1.36^.506^
***Household Dysfunction (HD)***				
Household Drug Use	765 (46.8%)	638 (47.4%)	416 (45.0%)	1.31^.519^
Witnessed IPV	467 (28.5%)	410 (30.4%)	224 (24.2%)	10.68^.005^
Household Mental Illness	757 (46.3%)	537 (39.9%)	303 (32.8%)	45.51***
Household Incarceration	419 (25.6%)	263 (19.5%)	129 (13.9%)	50.78***
Parental Divorce	1,076 (65.8%)	757 (56.2%)	384 (41.5%)	142.34***
**ACE Subclass**				
Child Abuse and Neglect Alone	88 (5.4%)	83 (6.2%)	96 (10.4%)	24.65***
Household Dysfunction Alone	304 (18.6%)	232 (17.2%)	172 (18.6%)	1.11^.58^
Both Child Abuse and Neglect and Household Dysfunction	1,046 (63.9%)	823 (61.1%)	475 (51.4%)	40.06***
**Lifetime Mood and Anxiety Disorder**				
Major Depressive Disorder	625 (38.2%)	557 (41.4%)	441 (47.7%)	21.86***
Bipolar Disorder	223 (13.6%)	238 (17.7%)	116 (12.5%)	14.34^.001^
General Anxiety Disorder	313 (19.1%)	206 (15.3%)	133 (14.4%)	12.39^.002^
**History of Suicide Ideation**				
Yes	373 (22.8%)	288 (21.4%)	228 (24.6%)	
No	1,263 (77.2%)	1,059 (78.6%)	697 (75.4%)	
Total	1,636 (37.2%)	1,347 (30.7%)	925 (21.1%)	3.337^.189^

**χ**^**2**^: value of the chi-squared test statistic;

^p^: p value.

***Significant at p <0.001.

### 3.2. ACEs and lifetime suicide ideation by generational cohort

Table 2 displays both the overall and generation-specific likelihood of having suicidal ideation by individual type of ACEs, controlling for mood and anxiety disorders, race/ethnicity, and gender. Upon examining the association of suicidal ideation with individual types of ACEs, the odds of having suicidal ideation were higher among all participants if they experienced: verbal abuse, physical abuse, sexual abuse, emotional neglect, physical neglect, witnessed domestic violence, household mental illness (*OR*s ranging from 1.25 to 1.72). Among Millennials, endorsing verbal abuse, sexual abuse, and/or emotional neglect were significantly associated with increased odds of suicide ideation (*OR*s ranging from 1.45 to 1.59). Similarly, Generation X were more likely to consider suicide if they experienced verbal abuse, sexual abuse, and/or emotional neglect (ranging from 1.45 to 1.81). The observations among Baby boomers was slightly different. Similar to Millennials and Generation X, Baby Boomer participants with a history of verbal abuse and/or emotional neglect were more likely to consider suicide. However, unique to Baby boomers were that additional ACEs, specifically physical abuse, physical neglect, parental divorce, witnessing intimate partner violence, and/or household mental illness, were significantly associated with higher odds of lifetime suicide ideation (*OR*s ranging from 1.42 to 1.88).

**Table 2:** Adjusted odds between ACE type and suicidal ideation^a^.

	All	Millennials	Generation X	Baby Boomers
ACE Type	OR (CI)^p^	OR (CI)^p^	OR (CI)^p^	OR (CI)^p^
***Child Abuse and Neglect (CAN)***				
Verbal Abuse	1.50 (1.30–1.74)***	1.45 (1.14–1.84)^.003^	1.45 (1.10–1.90)^.008^	1.75 (1.28–2.40)***
Physical Abuse	1.29 (1.11–1.49)***	1.26 (.99–1.60)	1.10 (.84–1.45)	1.55 (1.13–2.13)^.006^
Sexual Abuse	1.42 (1.22–1.66)***	1.52 (1.17–1.97)^002^	1.54 (1.16–2.04)^003^	1.42 (1.02–1.99)^041^
Emotional Neglect	1.72 (1.48–1.99)***	1.59 (1.25–2.02)***	1.81 (1.38–2.38)***	1.88 (1.37–2.57)***
Physical Neglect	1.32 (1.11–1.57)^.002^	1.31 (.98–1.74)	1.11 (.80–1.54)	1.82 (1.26–2.63)***
***Household Dysfunction (HD)***				
Household Drug Use	1.15 (.99–1.32)	1.23 (.97–1.57)	1.01 (.77–1.32)	1.34 (.97–1.83)
Divorce	1.12 (.97–1.30)	.94 (.73–1.21)	1.15 (.87–1.51)	1.45 (1.06–1.97)^.02^
Witnessed IPV	1.25 (1.07–1.46)^.006^	1.27 (.98–1.64)	.98 (.73–1.31)	1.65 (1.17–2.33)^.005^
Household Mental Illness	1.30 (1.12–1.51)***	1.26 (.99–1.60)	1.30 (.99–1.71)	1.50 (1.09–2.07)^.013^
Household Incarceration	1.16 (.98–1.38)	1.22 (.94–1.60)	.92 (.66–1.30)	1.30 (.85–1.99)

^a^Adjusted for mood and anxiety disorders, race/ethnicity, gender.

OR: Odd Ratio; CI: Confidence Interval;

^p^: p value.

***Significant at p <0.001.

### 3.3. Role of mood and anxiety disorders in association with ACEs and suicidal ideation

Table 3 shows the role of mood and anxiety disorders in association with ACEs (overall and composites-specific, CAN and HD) to suicidal ideation by generational cohort. Overall, endorsing >1 ACE(s) was found to have a significant effect on increased likelihood of suicidal ideation over and above the influence of mood and anxiety disorders, as well as gender and race/ethnicity. The test for trend showed an 8–14% increased risk for suicidal ideation when examining all generational cohorts. Additionally, the overlapping 95% CIs indicated that there was no significant difference in the ordinal ORs among the generational cohorts, even after controlling for mood and anxiety disorders, gender, and race/ethnicity. There was a significant relationship between the CAN composite of ACEs and suicidal ideation across all generations, even after controlling for the HD composite of ACEs, mood and anxiety disorders, race/ethnicity, and gender. In contrast, the relationship between the HD composite of ACEs was not found to be significantly associated with suicidal ideation, adjusting for both the CAN composite of ACEs, mood and anxiety disorders, race/ethnicity, and gender.

**Table 3:** Adjusted odds between any ACE or ACE composites, and suicidal ideation. CAN, child abuse and neglect; HD, household dysfunction.

	All	Millennials	Generation X	Baby Boomers
	OR (CI)^p^	OR (CI)^p^	OR (CI)^p^	OR (CI)^p^
**Any ACE**^a^	1.09 (1.06–1.13)***	1.09 (1.04–1.14)***	1.10 (1.04–1.15)***	1.14 (1.08–1.22)***
**CAN**^b^	1.18 (1.12–1.25)***	1.18 (1.08–1.30)***	1.2 (1.08–1.33)***	1.22 (1.09–1.36)***
**HD**^b^	1.00 (.95–1.07)	1.00 (.90–1.10)	.98 (.88–1.10)	1.06 (.93–1.20)

^a^Model includes mood and anxiety disorders, gender, and race/ethnicity.

^b^Model includes mood and anxiety disorders, gender, race/ethnicity, CAN, and HD.

OR: Odd Ratio; CI: Confidence Interval;

^p^: p value.

***Significant at p <0.001.

## 4. Discussion

Our study examined the well-established relationship between suicidal ideation and ACEs using a more diverse and low-income population than previous research in the field. An important discovery from this data is the outsized impact of emotional neglect on mood and suicidal ideation. Emotional neglect was a strong predictor of suicidal ideation overall. In contrast, prior research into this relationship did not identify emotional neglect as a significant predictor of suicidal ideation whereas it was a primary predictor in our research [31, 32]. Research on resiliency and social connectedness has shown the significance of having even one consistent and loving adult in a child’s life [33–35]. The results of our study point to the significant impact of lacking that support. Additionally, previous studies have assessed lifetime suicidality when correlating suicidality with ACEs. This study expands on our understanding of the temporal relationship of suicidality and ACEs by using the PHQ-9’s suicide question, which only reflects suicidality in the last two weeks. This relationship was notably missing from the seminal work examining the relationship between ACEs and suicidal ideation [25, 36]. While this is a novel and important feature of our research, it is also a limitation of that study as it means that some of the subjects may have experienced suicidal ideation in the past and not been identified in the study because the PHQ-9 question only inquires about the last two week.

Of particular interest is the association of ACEs with suicidal thoughts across generations. Dube et al. found no significant differences in the impact of ACEs across birth cohorts going back to 1900 leading them to conclude that the impact of ACEs remained stable in spite of external environmental changes related to the era people grew up in [27]. Our study found similarly that when ACEs were connected to suicide across generations, there was no meaningful difference in the odds ratio. However, in contrast, our study found significant differences in which individual ACEs were related to suicidal ideation by generation with certain types of ACEs (e.g., physical abuse, physical neglect, divorce, witnessing IPV, household mental illness) being predictors of suicidal ideation amongst baby boomers and not with other generations we assessed. Thus, the strong association between a specific type of ACEs and suicidal ideation found in baby boomers offers compelling evidence that explains the historically high rate of suicide among the boomer generation. When ACEs were viewed as composites of CAN or HD, the association with suicidal ideation was strongest for child abuse and neglect, across generations. Thus, the current study provides a more nuanced understanding of the association of ACEs with suicidal ideation by examining the impact of specific types of ACEs on different generational cohorts, which to our knowledge, had not been conducted in prior studies. It is difficult with the data we assessed to conclude why this is the case. Given the data is adjusted for race, ethnicity, and mood and anxiety disorders, it would be reasonable to conclude that these differences are explained by cultural factors including things like economic security differences between generations, the impact of technology on generations, or cultural changes in parenting across generations. This is an area ripe for future research.

Nonetheless, some limitations deserve comment. First, the study sample were patients seeking treatment for mental health or substance use disorders, and so are not likely to be representative of the general safety-net population. Second, the ACEs measure itself is limiting as it only captures whether or not a traumatic event has taken place, with no indication of how frequently or to what degree they experienced these events. Similarly, data regarding suicidality, mood, and anxiety disorders relied on patients’ self-reported data, which is subject to biases. Lastly, the study is limited by the potential of implicit bias on the part of healthcare professionals conducting the assessments, which could influence patients’ mental health diagnoses and subsequently, the conclusions made in this study.

Despite limitations, there are multiple significant implications for this data. First, when we evaluate patients for suicide risk, it is vital that we consider ACEs not just in terms of the total score but also in terms of the specific experiences or class of experiences of ACEs. While more total ACEs is more predictive of suicidal ideation we should pay attention even to patients with low ACE scores, particularly if they have experienced emotional neglect, verbal abuse, or sexual abuse. Second, ACEs seem to have differing impacts on generations, a novel finding in our study. For example, our study suggests that in older generations, several individual types of child abuse and neglect as well as household dysfunction were associated with lifetime suicidal ideation, while in younger generations, none of the individual ACEs related to household dysfunction were found to be associated with lifetime suicidal ideation. Community health centers and other primary care systems are trained to screen for suicidal ideation but ACEs as risk factors are rarely considered in screening [37]. When screening for suicidality we should also consider the way that suicidality and race/ethnicity interact and pay closer attention to the risk of household mental illness and intimate partner violence in communities of color and more attention to divorce and physical neglect in white communities. Lastly, additional research on the association of ACEs and suicidality in individuals not actively being managed in behavioral healthcare settings is also warranted.
